# Primary mucoepidermoid carcinoma of the liver with CRTC1-MAML2 fusion: a case report

**DOI:** 10.1186/s13000-019-0863-8

**Published:** 2019-07-27

**Authors:** Jiro Watanabe, Keita Kai, Ken Tanikawa, Mamoru Hiraki, Naohisa Mizukami, Shinichi Aishima, Takafumi Nakano, Hidetaka Yamamoto

**Affiliations:** 1 Departmant of Pathology, Yame General Hospital, Fukuoka, Japan; 2 Department of Pathology, Saga University Hospital, Nabeshima 5-1-1, Saga City, Saga, 849-8501 Japan; 3 Departmant of Surgery, Yame General Hospital, Fukuoka, Japan; 4 Departmant of Radiology, Yame General Hospital, Fukuoka, Japan; 5 Department of Pathology & Microbiology, Saga University Faculty of Medicine, Saga, Japan; 6 Department of Anatomic Pathology Graduate School of Medical Science, Kyushu University, Fukuoka, Japan

**Keywords:** Mucoepidermoid carcinoma, Liver, MAML2 fusion, Cholangiocellular carcinoma, Adenosquamous carcinoma

## Abstract

**Background:**

CRTC1-MAML2 fusion is often detected in low- or intermediate-grade salivary mucoepidermoid carcinoma (MEC), and it is associated with a favorable clinical course. Primary MEC of the liver is an extremely rare, aggressive tumor, and no study has investigated CRTC1-MAML2 fusion.

**Case presentation:**

A 79-year-old Japanese female presented with an approx. 5-cm hepatic mass lesion. We surgically resected the lesion under the clinical diagnosis of intrahepatic cholangiocarcinoma. The histological and immunohistochemical findings were consistent with high-grade MEC, consisting of squamoid, mucin-producing, and intermediate tumor cells. Our RT-PCR analysis revealed the presence of CRTC1-MAML2 fusion. This fusion gene was further confirmed by direct sequencing. The patient is still alive almost 10 years after the surgery.

**Conclusion:**

This is the first case report of primary MEC of the liver with CRTC1-MAML2 fusion, with long survival. The present case has significant implications for the entity of primary MEC of the liver which should be distinguished from adenosquamous carcinoma.

## Background

Mucoepidermoid carcinoma (MEC) is a common malignant neoplasm of the salivary glands but rarely arises in other organs, including the esophagus, pancreas, lung, breast, thymus, anal canal, lacrimal gland, thyroid gland, uterine cervix, and liver [1]. Primary MEC of the liver is extremely rare. Only 17 cases have been reported in the English literature [1]. Salivary MEC is often associated with chromosomal translocation, t (11;19)(q21;p13) [2], and this translocation generates a fusion gene comprised of the cAMP-regulated transcriptional co-activator 1 (CRTC1) at 19q21 and the mastermind-like gene 2 (MAML2) at 11q21 [3]. A fusion of CRTC3 at 15q26 and MAML2 is also reported as a specific fusion gene of salivary MEC [4].

As primary MEC of the liver is extremely rare, its clinicopathological features have been unclear. To the best of our knowledge, CRTC1/3-MAML2 fusion has not been investigated. We herein report the first case of primary MEC of the liver with CRTC1-MAML2 fusion.

## Case presentation

### Clinical summary

A 79-year-old Japanese female visited our hospital in complaining of right hypochondrium pain. She had no remarkable medical or family history. Laboratory tests on admission showed no abnormality except for elevated serum carcinoembryonic antigen (CEA) at 146 ng/mL (normal value, < 5.0) and carbohydrate antigen 19–9 (CA19–9) at 415 U/mL (normal value, < 37). Serum alpha-fetoprotein (AFP) and protein induced by Vitamin K absence or antagonists (PIVKA)-II were within the normal ranges, and hepatitis B viral antigen and hepatitis C antibody assays were both negative. Abdominal computed tomography (CT) revealed a mass lesion measuring approx. 5 cm in diameter at Segment 4 of the liver, and the mass lesion showed ring enhancement on a dynamic study. As positron emission tomography (PET)-CT showed no significant uptake signals other than the hepatic mass lesion, we ruled out the possibility of a metastatic liver tumor. Under the clinical diagnosis of intrahepatic cholangiocarcinoma, a left lobectomy and regional lymph node dissection were performed.

### Pathological findings

**Fig. 1:**
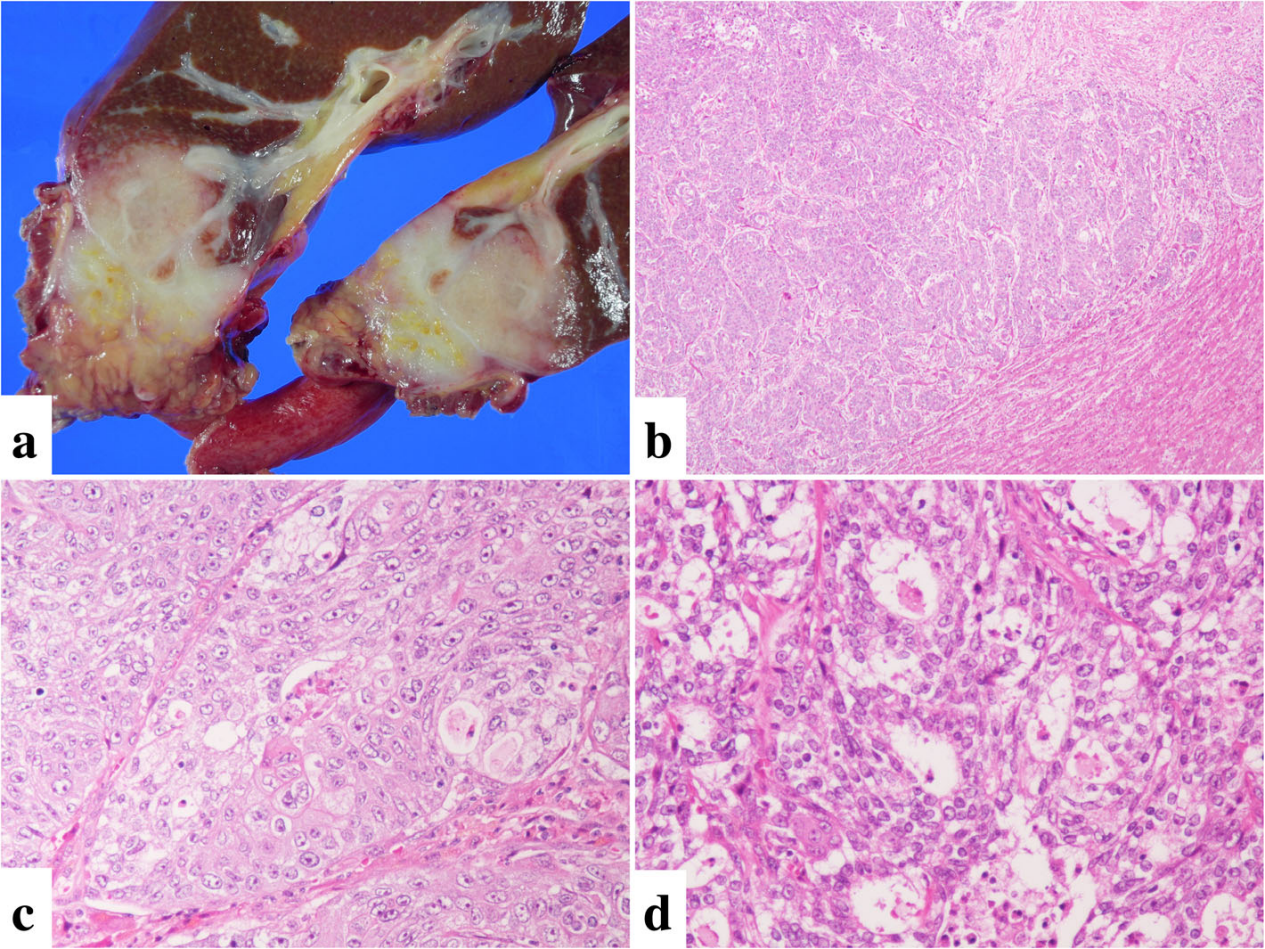
**a**: The cut surface of a resected specimen. A whitish solid tumor measuring 5.3 × 3.5 cm in maximal diameter was observed. The tumor showed infiltrative growth around the bile duct, and the border of the tumor was indistinct. The tumor directly invaded the omentum. **b-d:** Representative histological hematoxylin-eosin (HE) photographs of the tumor. **b:** The tumor showed invasive growth without capsule formation (HE × 40). **c:** The tumor partly showed squamous differentiation, including a sheet-like growth pattern, streaming nuclear arrangement, and mild keratinization. These tumor cells were considered squamoid cells. **d:** Although goblet-like cells were not apparent, a gland-like structure and intracytoplasmic and a small amount of extra-cytoplasmic mucin-producing cells were focally observed (HE × 200)

Grossly, the cut surface of the resected specimen showed an irregular, whitish solid tumor measuring 5.3 × 3.5 cm in maximal diameter. The border between the tumor and normal liver was indistinct, and the tumor directly invaded into the omentum and abdominal wall beyond the serosa of the liver (Fig. 1a).

Histologically, the tumor showed invasive growth without capsule formation (Fig. 1b). The background liver tissue showed an almost normal appearance. The tumor cells showed marked nuclear atypia and distinct nucleoli and had eosinophil-rich cytoplasm. Many mitotic Figs. (25/10 high-power field [HPF]), venous invasion, and intrahepatic metastatic lesion were observed. Lymphatic vessel invasion and lymph node metastasis were not apparent. Although overt keratinization was not apparent, nests of squamoid cells showing squamous differentiation were seen (Fig. 1c). We did not observe goblet-like mucin-producing cells, but a gland-like structure and intracytoplasmic and a small amount of extra-cytoplasmic mucin-producing cells were focally observed (Fig. 1d). Based on these morphological features, we considered the pathological diagnosis of high-grade mucoepidermoid carcinoma predominantly composed of an intermediate cell component.

**Fig. 2:**
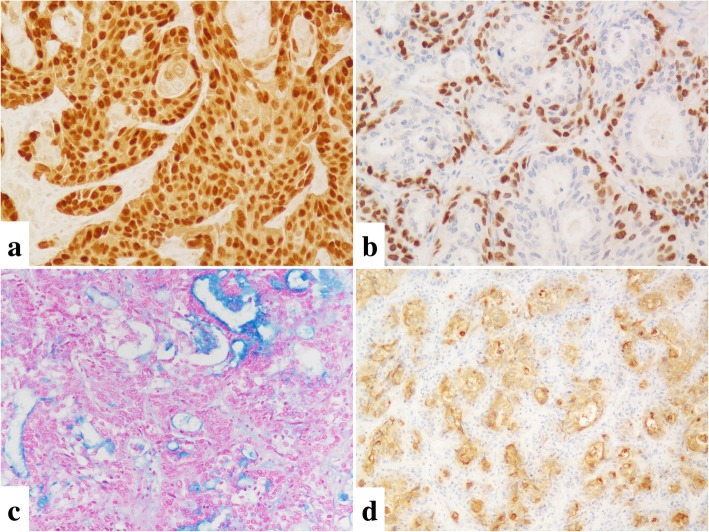
**a**: Squamoid and intermediate cells were positive for both p63 (nucleus) and CK14 (cytoplasm) by double immunostaining of p63 and CK14 (× 200). **b:** Mucin-producing cells were negative for p63 (× 200). **c:** Alcian blue staining highlights the mucin of mucin-producing cells (× 200). **d:** Tumor cells focally positive for CEA (× 200)

In immunohistochemistry, the tumor cells were positive for cytokeratin (CK) 7 and CK19 but negative for CK8 and hepatocyte paraffin-1. Squamoid cells and intermediate cells were positive for p63, CK14 (Fig. 2a), CK5/6, and involucrin. Mucin-producing cells were negative for p63 (Fig. 2b) but highlighted by both alcian blue (Fig. 2c) and mucicarmine staining. The tumor cells were focally positive for CEA (Fig. 2d) and CA19–9. Based on these findings, we made the final diagnosis of primary MEC of the liver.

**Fig. 3:**
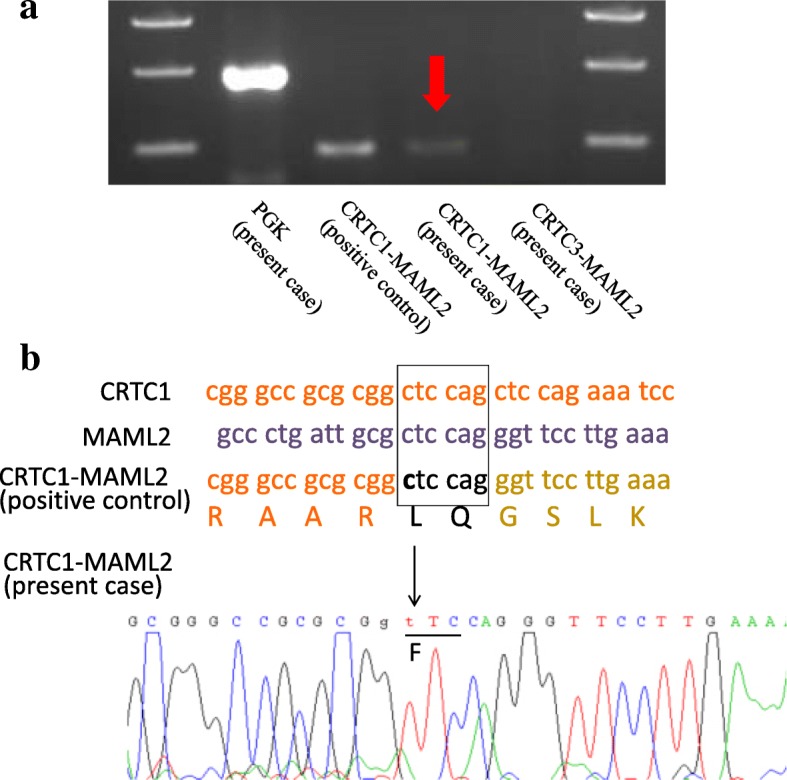
Results of the CRTC1/3-MAML2 fusion gene analysis. **a:** The RT-PCR products which electrophoresed in 2% agarose gel showed the presence of CRTC1-MAML2 fusion (*red arrow*). **b:** CRTC1-MAML2 fusion was confirmed by direct sequencing

For the analysis of CRTC1/3-MAML2 fusion gene, total RNA was isolated from paraffin-embedded tissue using the miRNeasy FFPE Kit (Qiagen, Valencia, CA, USA), and first-strand cDNA was synthesized using Superscript III Transcriptase (Invitrogen, Carlsbad, CA). The detection of CRTC1/3-MAML2 fusion was performed by reverse transcription-polymerase chain reaction (RT-PCR) using the described primers and conditions [5]. The sequence of the PCR product was confirmed by direct sequencing methods using an ABI Prism 310 sequence analyzer (Applied Biosystems, Foster City, CA). The RT-PCR products of the patient which electrophoresed in 2% agarose gel showed the presence of CRTC1-MAML2 fusion (Fig. 3a). CRTC1-MAML2 fusion was confirmed by direct sequencing (Fig. 3b).

### Clinical course

As adjuvant therapy, the oral administration of S-1 (100 mg/day) was started. Two years after the patient’s surgery, para-aortic lymph node swelling was detected by abdominal CT although serum tumor markers (CEA and CA19–9) were within normal ranges. Under the clinical diagnosis of suspected lymph node recurrence, radiation therapy (total 60 Gy) combined with S-1 therapy (100 mg/day) was performed for 13 months. No further therapy was performed. At the time of this writing, almost 10 years after the surgery, the patient is alive with no evidence of tumor recurrence.

## Discussion and conclusions

Primary MEC of the liver was first reported by Pianzola and Drut in 1971 [6]. Several authors speculated that hepatic MEC may arise from the terminal bile duct in association with squamous metaplasia [6–8]. Some authors proposed that MEC of the liver might originate from a congenital cyst [9, 10]. However, the etiology and pathogenesis of hepatic MEC remains unclear. To the best of our knowledge, no previous hepatic MEC series investigated the CRTC1/3-MAML2 fusion status.

In salivary MECs, CRTC1-MAML2 fusion was detected in approx. 40% of low- or intermediate-grade MECs and was associated with favorable clinicopathological features and an indolent clinical course [11]. Salivary MECs with CRTC3-MAML2 fusion are also considered indolent tumors with a favorable prognosis [4]. Based on an array CGH (comparative genomic hybridization) study of genomic imbalances, Jee et al. [12] proposed the following subclassification of MECs according to the CRTC1-MAML2 fusion status: (a) low-grade, fusion-positive tumors with no or few genomic imbalances and favorable prognosis, (b) high-grade, fusion-positive tumors with multiple genomic imbalances and unfavorable prognosis, and (c) a heterogeneous group of high-grade, fusion-negative non-MEC adenocarcinomas with multiple genomic imbalances and unfavorable outcomes.

MEC of the liver is an aggressive tumor with a poor prognosis irrespective of the surgical treatment, most patients with hepatic MEC have died within 6 months after the initial diagnosis [1, 13]. However, our patient’s case followed a favorable clinical course with 10 years’ survival despite the high-grade morphology, locally aggressive tumor with vascular invasion and direct invasion of the omentum and abdominal wall, and intrahepatic metastasis. This clinical course was distinctly different from those described in previous series. We speculate that hepatic MEC with MAML2 fusion is also associated with a favorable prognosis, the same as salivary MECs. However, the confirmation of this is not possible because no previous study of a hepatic MEC case investigated MAML2 fusion. Further investigations of the MAML2 fusion status in hepatic MEC series are clearly needed.

The pathological diagnosis of MEC is based on the presence of squamoid, mucin-producing, and intermediate tumor cells. However, because of this tumor’s rarity, the pathological diagnosis of hepatic MEC is not easy. Several authors have warned of the risk of misdiagnosis of hepatic MEC as cholangiocarcinoma with squamous metaplasia, adenosquamous carcinoma, or squamous cell carcinoma [1, 14]. The question of whether MEC of the liver is a distinct entity that should be distinguished from adenosquamous carcinoma remains to be answered. In addition, although it was excluded by PET-CT in this case, the possibility of hepatic metastases of MEC from salivary gland or potentially other organ should be always considered [15, 16].

Saeki et al. [17] investigated CRTC1/3-MAML2 fusion status in 16 cases of morphologically distinct pancreatic MEC (Pan-MEC), and they reported that all of the patients in the series had no MAML2 fusion. Since the clinicopathological features, survival, and immunohistochemical features of Pan-MEC are not significantly different from those of pancreatic adenosquamous carcinoma cases, Saeki et al. proposed the terminology of ‘pancreatic adenosquamous carcinoma with MEC-like features.’ However, we have fortunately encountered a case of hepatic MEC with MAML2 fusion despite hepatic MEC is even more rare than Pan-MEC. This fact implies that hepatic MEC is essentially different from adenosquamous carcinoma. We therefore consider that the implications of the present case for the entity of hepatic MEC are quite significant.

In conclusion, we have provided the first report of a case of primary MEC of the liver with CRTC1-MAML2 fusion and long-term survival. Our patient’s case has significant implications for this entity. A further accumulation of cases and investigations is required to clarify the frequency of the MAML2 fusion and its clinicopathological characteristics in primary MEC of the liver.

## Data Availability

Please contact corresponding author for data requests.

## Abbreviations

CRTC1: cAMP-regulated transcriptional co-activator 1
MAML2: Mastermind-like gene 2
MEC: Mucoepidermoid carcinoma
